# Assessment of Functional Status of Human Leukocyte Antigen Class I Genes in Cancer Tissues in the Context of Personalized Neoantigen Peptide Vaccine Immunotherapy

**DOI:** 10.1200/CCI-24-00174

**Published:** 2025-07-02

**Authors:** Vijay G. Padul, Nupur Biswas, Mini Gill, Jesus A. Perez, Javier J. Lopez, Santosh Kesari, Shashanka Ashili

**Affiliations:** ^1^ Rhenix Lifesciences, Hyderabad, India; ^2^ CureScience, San Diego, CA; ^3^ Medicine, Pan American Cancer Treatment Center, Tijuana, Mexico; ^4^ Neurology, Pan American Cancer Treatment Center, Tijuana, Mexico; ^5^ Department of Translational Neurosciences, Pacific Neuroscience Institute and Saint John's Cancer Institute at Providence Saint John's Health Center, Santa Monica, CA

## Abstract

**PURPOSE:**

Accurate human leukocyte antigen (HLA) typing is an essential step for designing peptide vaccines used in the personalized neoantigen peptide vaccine immunotherapy (PNPVT) in patients with cancer. The reasons for variation in the patient response to PNPVT are yet unknown. One of the reasons could be the somatic changes in the HLA genes in the cancer cells. The objective of the present research was to analyze the somatic status of HLA class I genes in cancer tissue through integrative genomic analysis and to identify high-confidence subset of potentially functional cancer somatic HLA class I genotype relevant to PNPVT.

**PATIENTS AND METHODS:**

Whole-exome (paired tumor-normal) and RNAseq (tumor) paired-end sequencing data from 24 patients with cancer were used for the analysis. The genotyping of HLA class I was performed using four HLA typing software tools. To assess the functional status of HLA class I genes in the cancer tissue, we analyzed somatic mutation, HLA gene loss of heterozygosity, and chromosome 6 copy loss status in cancer exome data.

**RESULTS:**

Somatic mutations in HLA genes were detected in the tumor data of five patients, and somatic HLA gene loss of heterozygosity was identified in the tumor data of five patients. Complete or partial chromosome 6 copy loss was detected in eight patient samples.

**CONCLUSION:**

The results indicate that HLA class I genes may get affected by somatic changes in cancer tissue, and assessment of the somatic status of the HLA genotype should be performed in the cancer tissues. The results provide robust rational for removal of mutated or lost HLAs from the personalized neoantigen peptide prediction pipeline to potentially increase the efficacy of the PNPVT. Further functional studies are needed to assess the impact of HLA gene mutations/loss on PNPVT outcomes.

## INTRODUCTION

The major histocompatibility complex (MHC) molecules play a key role in T-cell–mediated self- and non–self-discrimination by the adaptive immune system in response to infectious diseases and cancer.^1-5^ In humans, the MHC system is known as human leukocyte antigen (HLA) and is located on the short arm of chromosome 6 on band 6p21.3. The classical class I genes encoded in the HLA region are *HLA-A*, *HLA-B*, and *HLA-C*, whereas the class II region encodes *HLA-DPA1*, *HLA-DPB1*, *HLA-DQA1*, *HLA-DQB1*, *HLA-DRA*, and *HLA-DRB1* genes.^6^ The HLA genes are codominantly inherited from parents, and both the alleles of the gene are expressed on the cell surface. The HLA gene cluster is highly polymorphic, and new alleles of HLA genes are continuously being discovered in the human population.^7,8^ The IPD-IMGT/HLA Database is a repository of highly curated HLA sequences. By the end of 2024, the database hosted sequences for 42,026 alleles of the HLA system genes.^9^

**CONTEXT**

**Key Objective**
To analyze the somatic status of human leukocyte antigen (HLA) class I genes in cancer tissues through integrative genomic analysis and to identify high-confidence subset of potentially functional cancer somatic HLA class I genotype relevant to personalized neoantigen peptide vaccine immunotherapy (PNPVT).
**Knowledge Generated**
In cancer cells, the HLA genes may get affected because of somatic changes such as mutation, HLA gene copy loss, or entire chromosome 6 loss, which may affect the HLA function. Further study of the potential adverse impact of such changes on PNPVT outcomes is needed. The HLA gene somatic status assessment should be incorporated in the clinical workflow of PNPVT pipelines.
**Relevance *(Z. Bakouny)***
With personalized neoantigen vaccines being tested across types of tumors, the accurate evaluation of HLA type is becoming increasingly important. In this study, the authors suggest that tumor-based HLA typing should be used instead of germline HLA typing during neoantigen vaccine design in order to more accurately reflect antigen presentation in tumors and therefore improve neoantigen vaccine design.*
*Relevance section written by *JCO Clinical Cancer Informatics* Associate Editor Ziad Bakouny, MD, MSc.

HLA genotyping is a significant step in cancer antitumor immunotherapy, which uses personalized neoantigen peptide molecules predicted to bind to HLA proteins.^10-12^ Personalized neoantigen peptide vaccine immunotherapy (PNPVT) focuses on training the adaptive immune system of the patient by exogenously introducing neopeptides into the patient body.^13-17^ The adequate binding of peptide to HLA protein is dependent on the peptide sequence and the type of HLA allele present in the patient.^18-21^ To make an accurate prediction of personalized neoantigen peptide sequences for a patient with cancer, accurate HLA genotyping of the patient is needed.^17,22-24^ For personalized neoantigen peptide discovery and prioritization, three types of sequencing data from the patient with cancer are needed, which are tumor exome, tumor RNAseq, and normal exome.^5,25^ These three sequencing sources can also be used for deciphering the patient HLA genotype. The choice of normal tissue– or cancer tissue–derived HLA genotype for personalized neoantigen peptide prediction could potentially be an important factor in the success of PNPVT. In current practice, normal tissue–derived HLA genotype is used for personalized neoantigen peptide prediction.^5^ Neoantigen processing, loading on the HLA protein, and presentation on the extracellular surface take place inside the cancer cell.^1^ The cancer cells are subject to somatic changes in their genome as a result of somatic mutations^26^ and somatic chromosome copy number alterations,^27^ which, if present in HLA genes, may potentially serve as an immune evasion mechanism/adaptation by cancer cells.^27,28^ This necessitates probing of the somatic status of the HLA genotype in the cancer tissue of the patient. An integrative genomics approach for identification of somatic HLA gene mutations, chromosome 6 copy loss, and HLA allele loss of heterozygosity will be helpful to determine the HLA genotype status in the cancer tissue. Furthermore, designing the personalized neoantigen peptide vaccines against only the intact HLA proteins may also be helpful to increase the precision of the PNPVT.

The high polymorphism of HLA genes makes accurate genotyping of HLA alleles particularly challenging.^29^ Many benchmarking studies have found the accuracy of various HLA typing tools to be variable,^26,30-34^ with no single tool consistently outperforming the others.^32^ With this limitation, if a single software tool and a single sequencing source are used for HLA genotyping, the predicted HLA genotype may be unreliable. One way to overcome this limitation could be the use of multiple HLA typing software packages on multiple data sources from the same individual. Numerous studies have benchmarked HLA typing algorithms using high-throughput sequencing data^30,32-36^ and multiple sequencing sources from the same individual. However, to our knowledge, no study has compared HLA genotype outcomes using three sequencing sources (normal exome, tumor exome, and tumor RNAseq) from the same individual.^37,38^

**FIG 1.**
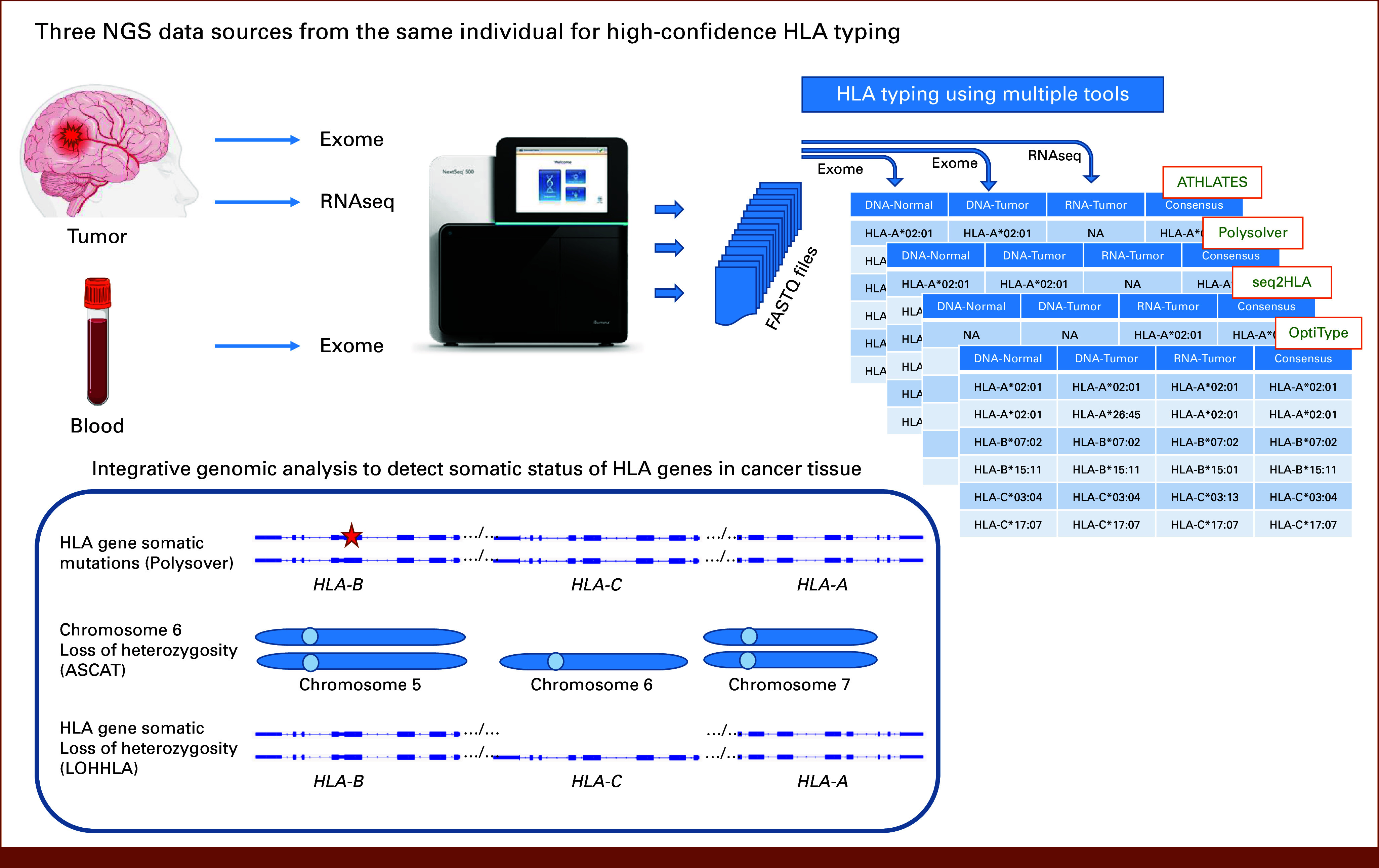
Analysis workflow describing the use of three sequencing data sources from the same individual with four HLA typing software packages combined with detection of somatic mutations and HLA loss of heterozygosity to generate a high-confidence HLA genotype subset. HLA, human leukocyte antigen; NA, not available; NGS, next generation sequencing.

In this article, we present analysis to identify therapeutically significant HLA genotype subsets by using an ensemble of four software tools, OptiType,^39^ Polysolver,^26^ ATHLATES,^40^ and seq2HLA^41^ for HLA class I typing using three types of sequence sources from the same individual, that is, tumor tissue exome, normal tissue exome, and tumor tissue RNAseq. We further present an integrative genomic analysis of HLA genes in cancer tissues to identify a subset of functional HLA genotype relevant to PNPVT. The overall analysis workflow is shown in Figure 1. The HLA typing results from these four software packages and three tissue sources were used to find the most frequent or consensus two-field^42^ HLA allele for a given HLA gene. The higher the frequency of HLA call for the predicted HLA allele using multiple data sources by multiple tools, the higher will be the confidence in the predicted HLA allele for inclusion in the HLA genotype for the individual. In cancer cells, the HLA genes may be subject to somatic mutations or allele copy loss.^26,27^ To assess the status of HLA class I genes in the cancer tissue, we analyzed somatic mutation status, HLA gene loss of heterozygosity, and chromosome 6 copy loss in cancer exome data. HLA allele loss of heterozygosity detection analysis was performed using LOHHLA^27^ which is the only software tool available for HLA class I allele loss of heterozygosity detection. LOHHLA uses four-field HLA genotype–based exact HLA gene nucleotide sequences for alignment and subsequent coverage analysis of tumor exome and normal exome HLA reads.^27^ We used ASCAT^43,44^ to detect B-allele frequency (BAF)–based chromosome copy alterations present in the tumor exome samples and to determine tumor purity and ploidy estimates required by LOHHLA software.

## PATIENTS AND METHODS

### Samples and Data

Whole-exome (paired tumor-normal) and RNAseq (tumor) paired-end Illumina sequencing data from 24 patients with cancer were used for the analysis. Formalin-fixed paraffin-embedded tumor tissue was used for tumor exome and tumor RNAseq sequencing, and peripheral blood was used for normal exome sequencing.

The data were available from the following patients with cancer: eight patients with glioblastoma (GBM), three patients with chordoma, two patients with esophageal carcinoma (ESCA), two patients with high-grade glioma (HGG), two patients with low-grade glioma, two patients with lung squamous cell carcinoma (LUSC), one patient with breast carcinoma, one patient with liver hepatocellular carcinoma (LIHC), one patient with stomach adenocarcinoma (STAD), one patient with sarcoma (SARC), and one patient with miscellaneous (MISC) cancer. The read length of all exome data was 126 bp (except for GBM_E, for which the exome and RNAseq data read length was 101 bp), and the read length of all RNAseq was 76 bp. All samples were obtained under written informed consent with Institutional Review Board approval. The data quality was assessed using Picard,^45^ FastQC,^46^ and Qualimap.^47^ HLA typing information obtained by any other method was not available for any of the samples studied.

### Somatic Mutations and Loss of Heterozygosity in HLA Genes

Somatic mutations in the HLA class I genes were assessed using Polysolver^26^ which compared the four-field HLA genotype sequences for each HLA class I gene from tumor exome samples with those from normal exome samples using Mutect2.^48^ The normal four-field HLA genotype used for the analysis was deduced using normal exome data with Polysolver. The chromosome level loss of heterozygosity was analyzed using ASCAT^44^ which predicts chromosomal copy number alteration by analyzing the BAF profile of tumor and normal exome data. The tumor purity, tumor ploidy estimates by ASCAT, and normal sample–based four-field HLA genotype deduced from Polysolver were used to analyze the loss of heterozygosity (allele-specific loss) status of HLA class I genes using LOHHLA.^27^

### HLA Typing

HLA typing was performed using four tools: ATHLATES, OptiType, Polysolver, and seq2HLA. ATHLATES and Polysolver use exome sequencing data as input data. Seq2HLA uses RNAseq data as input, whereas OptiType can use both exome/whole-genome and RNAseq data for HLA typing. ATHLATES was used with Novoalign (Novocraft^49^) aligner to align the FASTQ reads to the reference HLA database. OptiType^39^ was used with RazerS3^50^ aligner for read alignment with eight threads and CBC integer linear programming solver. Polysolver uses the human genome–aligned binary alignment map (BAM) files as input. For generating BAM files, the exome FASTQ files were aligned to human reference genome hg38 using BWA MEM aligner^51^ with default parameters. The BAM files were deduplicated using Picard.^45^ Polysolver^26^ was used with Novoalign (Novocraft^49^) aligner for mapping the FASTQ reads extracted from input BAM files. Seq2HLA was used with Bowtie^52^ aligner for read mapping. The HLA typing resolution of ATHLATES and Polysolver was four-field resolution, whereas for OptiType and seq2HLA, it was two-field resolution. All the HLA allele types generated by the four tools were normalized into two-field resolution for the comparative analysis as two-field resolution is the most relevant resolution for neoantigen peptide prediction. For each HLA allele genotype, the consensus HLA genotype call was determined as the unanimous or most frequent two-field HLA type call from among the eight HLA genotype calls deduced by four software packages using three sequencing data sources.

### Ethical Statement

All samples were obtained under written informed consent with Institutional Review Board approval.

## RESULTS

### Data Quality

The quality assessment of exome and RNAseq data is presented in the Data Supplement (Fig S1). Exome sequencing coverage, ranging from 1× to 51×, was assessed using Qualimap and is shown in the Data Supplement (Figs S1A and S1B). Sequence bases with a Phred quality score above 20 ranged from 93.03% to 99.09% (Data Supplement, Figs S1C-S1E). The mean depth of exome sequencing coverage estimated using Qualimap, for normal exome (21.7× to 457×) and tumor exome (25.8× to 396×) samples, is presented in the Data Supplement (Fig S1F). The exome sequencing error rates estimated using Qualimap, for normal exome and tumor exome samples, are shown in the Data Supplement (Fig S1G). The Data Supplement (Fig S2A) shows normalized RNAseq gene coverage for all 24 samples with similar gene coverage patterns across samples with 5’ bias but no significant degradation. The Data Supplement (Fig S2B) shows RNAseq data read counts for all 24 samples, which ranged from 33.06 million to 187.95 million FASTQ reads.

### HLA Genotyping

**FIG 2.**
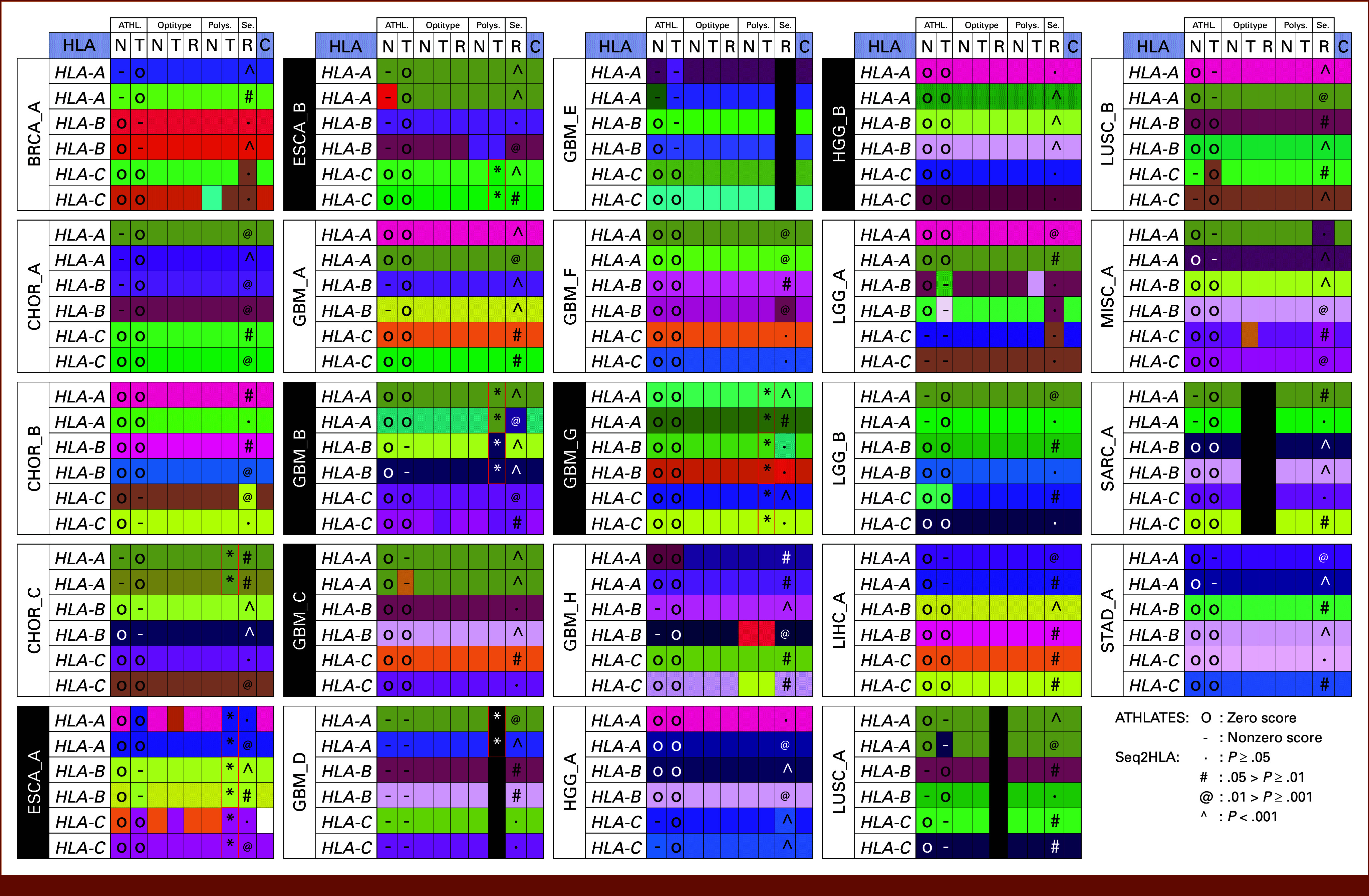
HLA class I typing results for 24 patients derived from using original FASTQ of normal-DNA exome (N), tumor-DNA exome (T), and tumor-RNA RNAseq (R) using four software tools (ATHLATES [ATHL.], OptiType, Polysolver [Polys.], seq2HLA [Se.]). Each distinct two-field HLA call is masked with a distinct color shade. The cells with black color indicate that HLA typing did not work for the given HLA allele. The sample names highlighted with black cells indicate that chromosome 6 copy loss was observed in the corresponding tumor tissue. The cells labeled with * are the HLA alleles for which statistically significant loss of heterozygosity was detected. The quality metrics from the respective HLA typing tool for HLA genotypes are overlaid on respective HLA genotype cells from ATHLATES and seq2HLA. Polysolver and OptiType results are not accompanied by any quality metrics. BRCA, breast carcinoma; CHOR, chordoma; ESCA, esophageal carcinoma; GBM, glioblastoma; HGG, high-grade glioma; HLA, human leukocyte antigen; LGG, low-grade glioma; LIHC, liver hepatocellular carcinoma; LUSC, lung squamous cell carcinoma; MISC, miscellaneous; SARC, sarcoma; STAD, stomach adenocarcinoma.

HLA genotyping results for HLA class I using four software tools ATHLATES, OptiType, Polysolver, and seq2HLA are shown in Figure 2. All distinct two-field HLA calls were masked with distinct color shades for visualization in Figure 2. OptiType and Polysolver do not provide any specific quality metrics with the final HLA genotype output. ATHLATES provides a score value of zero for reliably constructed allelic pairs, whereas nonzero score reflects incomplete capturing of full-length HLA gene. Most of the ATHLATES HLA class I genotype results were accompanied by a score value of zero, indicating reliably constructed allelic pairs during HLA typing and good-quality HLA calls (Fig 2). The software seq2HLA provides *P* values for each HLA genotype call. A majority of HLA class I genotype results by seq2HLA were accompanied by statistically significant *P* values (*P* < .05). The quality metrics for HLA genotyping results using ATHLATES and Seq2HLA are overlaid on respective HLA genotype calls in Figure 2. In many samples, the same HLA allele type is seen to be called by different software tools using different tissue data sources. Consistent HLA genotypes especially from DNA exome and RNAseq data represent the high-confidence HLA genotype for the given patient. Unanimous HLA typing outcomes were seen in 79.17% (114 of 144) of results (Fig 2) from four software tools and three sequencing sources. The best performance was shown by OptiType with 96.96% unanimous HLA typing results using normal exome, tumor exome, and tumor RNAseq data. All HLA typing results from OptiType derived using normal exome and tumor RNAseq were found to be same (Table 1). One reason for HLA genotype mismatch was found to be HLA gene loss of heterozygosity and chromosome 6 copy loss. HLA typing with OptiType did not work for samples LUSC_A (RNA) and SARC_A (T, R), Polysolver did not work for sample GBM_D (T), and seq2HLA did not work for sample GBM_E (R) because of undetermined reasons (shown in black color in Fig 2).

**TABLE 1.** Comparison of HLA Typing Results

Sample Groups for Comparison of HLA Typing Results			Samples With HLA Typing Results	Same HLA Typing Results, %	Same HLA Typing Results	All HLA Typing Results	All HLA Typing Mismatches	HLA Typing Mismatch Because of Chromosome 6 Copy Loss (LOH)
OptiType								
N	T	R	22	96.96	128	132	4	3
N	T		23	97.10	134	138	4	3
N		R	22	100.00	132	132	0	0
	T	R	22	96.96	128	132	4	3
ATHLATES								
N	T		24	92.36	133	144	11	3
Polysolver								
N	T		23	92.75	128	138	10	8

NOTE. The table shows the comparison of HLA typing results using software tools OptiType, ATHLATES, and Polysolver when normal exome (N), tumor exome (T), and tumor RNAseq (R) data were used for HLA genotyping analysis.

Abbreviations: HLA, human leukocyte antigen; LOH, loss of heterozygosity.

### HLA Gene Somatic Mutations and Loss of Heterozygosity

Somatic mutations were found to be present in five patient tumor data, that is, ESCA_A, GBM_E, GBM_H, LIHC_A, and STAD_A. The HLA alleles with somatic mutations are described in Table 2. Of the five mutations, four were found to be present in the intronic region, whereas one was found to be affecting the exonic region. The mutation in exon 4 of HLA-A*01:01:01:01 in the ESCA_A sample resulted in missense amino acid change p.A270S. This amino acid change is classified as likely benign or benign by UniProtKB/Swiss-Prot.^53^ The Integrated Genomic Viewer snapshots of the FASTQ read alignments to reference allele from normal and tumor tissues around HLA somatic mutations are shown in the Data Supplement (Fig S3). The HLA gene mutations could not be validated by an alternative method because of the lack of availability of biological samples for the analysis.

**TABLE 2.** HLA Gene Mutations

Sample	HLA	Position	Ref Allele	Alt Allele	Tumor Supporting Reads (total-Q20:ref:alt)	Normal Supporting Reads (total-Q20:ref:alt)	Region	Exon Number | Protein Amino Acid Change
ESCA_A	A*01:01:01:01	2,058	G	T	12:8:4	161:165:0	Exon	4 | p.A270S
GBM_E	C*15:02:01:01	367	G	A	214:10:205	188:189:0	Intron	—
GBM_H	A*34:01:01:01	2,494	A	G	16:14:5	22:22:1	Intron	—
LIHC_A	A*68:02:01:01	3,055	G	C	55:47:8	69:69:0	Intron	—
STAD_A	A*68:01:02:01	3,055	G	C	19:16:3	31:32:0	Intron	—

NOTE. Somatic mutations identified in the HLA genes in exome sequencing data from cancer tissue samples.

Abbreviations: ESCA, esophageal carcinoma; GBM, glioblastoma; HLA, human leukocyte antigen; LIHC, liver hepatocellular carcinoma; STAD, stomach adenocarcinoma.

**FIG 3.**
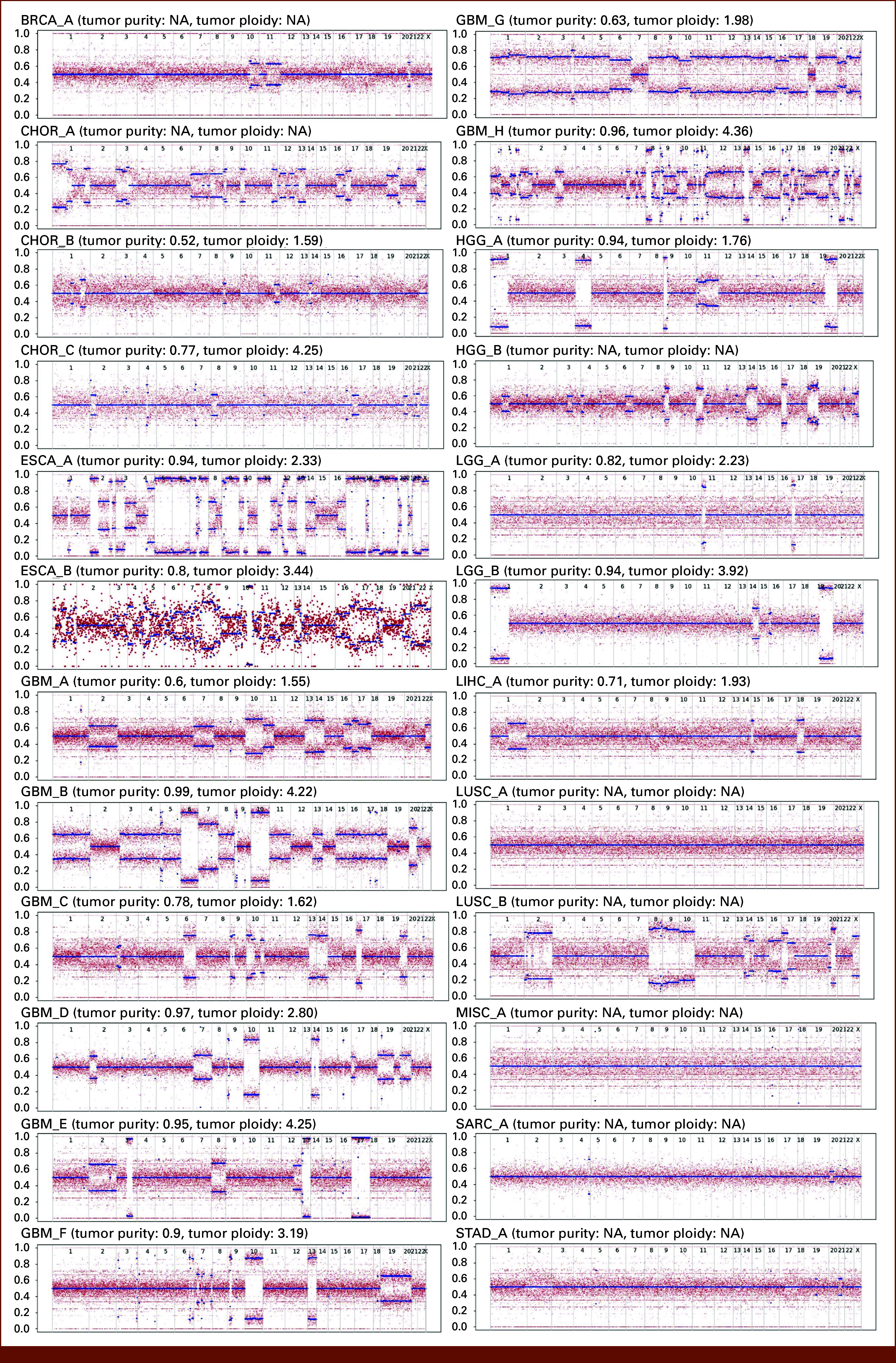
Chromosome (*X*-axis) copy loss detected by the BAF profiles of the 24 tumor samples. BAF on the *Y*-axis shows the relative presence of each of the two alternative nucleotides (called A and B) at each profiled heterozygous SNP locus. BAF deviating from 0.5 indicates chromosome copy loss. BAF, B-allele frequency; BRCA, breast carcinoma; CHOR, chordoma; ESCA, esophageal carcinoma; GBM, glioblastoma; HGG, high-grade glioma; LGG, low-grade glioma; LIHC, liver hepatocellular carcinoma; LUSC, lung squamous cell carcinoma; MISC, miscellaneous; NA, not available; SARC, sarcoma; SNP, simple nucleotide polymorphism; STAD, stomach adenocarcinoma.

BAF-based complete loss of heterozygosity of chromosome 6 was observed in samples ESCA_A, ESCA_B, GBM_B, and GBM_G, whereas partial chromosome 6 copy loss was observed in GBM_C, GBM_F, GBM_H, and HGG_B (Fig 3). Statistically significant somatic HLA class I allele loss of heterozygosity was found in five patient tumor data (Table 3). The loss of heterozygosity of HLA class I alleles was also seen in the HLA genotype calls by Polysolver, and these are highlighted with an asterisk in Figure 2. HLA loss of heterozygosity was reflected in the HLA genotype from tumor samples with high tumor purity (Figs 2 and 3). The high-confidence subset of functional somatic HLA genotype in cancer cells could be obtained by excluding the HLA alleles affected by somatic mutations which may potentially affect the HLA protein structure and function, by excluding HLA genes lost because of HLA loss of heterozygosity and excluding HLA genes located on lost copy of chromosome 6.

**TABLE 3.** Somatic HLA Gene Loss of Heterozygosity in Tumor Samples

Tumor	LossAllele	KeptAllele	PVal_unique	UnPairedPval_unique	numMisMatchSitesCov	propSupportiveSites
CHOR_C	A*32:01	A*02:1040	1.920E-02	2.568E-02	53	88.68
ESCA_A	A*01:01	A*68:02	1.185E-25	1.892E-28	78	100.00
ESCA_A	B*35:02	B*14:02	4.060E-18	9.062E-21	61	100.00
ESCA_A	C*04:01	C*08:02	1.907E-16	7.499E-19	38	100.00
ESCA_B	C*03:03	C*07:02	3.731E-02	9.348E-02^a^	62	58.06
GBM_B	A*33:01	A*02:01	3.931E-17	2.232E-25	53	100.00
GBM_B	B*14:02	B*18:01	1.117E-13	1.196E-20	48	100.00
GBM_D	A*11:01	A*02:01	2.185E-04	2.265E-03	80	85.00
GBM_G	A*23:01	A*01:02	6.043E-04	6.324E-04	66	100.00
GBM_G	B*35:03	B*49:01	4.644E-04	6.279E-04	41	100.00
GBM_G	C*12:03	C*07:01	1.477E-03	3.311E-03	61	100.00
LGG_A	B*40:02	B*35:01	3.281E-02	6.780E-02^a^	55	52.73
LGG_B	B*52:01	B*58:01	3.829E-02	5.645E-02^a^	25	96.00

NOTE. The column table labels are as follows (as provided in the LOHHLA output): LossAllele: HLA allele that is present at lower frequency and which is potentially subject to loss, KeptAllele: HLA allele that is present at higher frequency, which is potentially not subject to loss, PVal_unique: paired *t*-test *P* value related to the difference in logR between two alleles, ensuring that each read only contributes once. UnPairedPval_unique: unpaired *t*-test *P* value related to the difference in logR between two alleles, ensuring that each read only contributes once, numMisMatchSitesCov: number of mismatch sites with sufficient coverage, propSupportiveSites: proportion of mismatch sites that are consistent with loss or allelic imbalance.

Abbreviations: CHOR, chordoma; ESCA, esophageal carcinoma; GBM, glioblastoma; HLA, human leukocyte antigen; LGG, low-grade glioma.

a Not statistically significant.

## DISCUSSION

The results from the present study showcase the utility of using three sequencing sources from patients with cancer for determining the high-confidence HLA genotype. Integrative genomic analysis using these data sources can lead to identification of HLA gene somatic mutations, HLA gene loss of heterozygosity, and chromosome 6 copy loss. Findings from each of these steps provide significant information about the potentially functional HLA genotype in the cancer tissue. We did not find any other study which reported HLA typing from these three high-throughput sequencing data sources from a single individual. The superiority of ensemble HLA calling using multiple tools has been shown previously.^30,31^ A study with HLA typing using single data source and a combination of six tools showed high accuracy for unanimous calls.^54^ The ensemble HLA genotyping approach may also help to avoid error patterns associated with the specific tool and to generate reliable HLA typing results.^30^ We did not observe any correlation of sequencing coverage and consistency of HLA typing results across tissue types and tools. The sample ESCA_B has the lowest sequencing mean coverage (normal exome 21.73× and tumor exome 25.88×, Data Supplement, Fig S1F), but the HLA typing results are consistent across tools except one mismatch (*HLA-A*, ATHLATES) and other two mismatches because of loss of heterozygosity (LOH) as an effect of chromosome 6 copy loss (*HLA-B*, *HLA-C* by Polysolver; Fig 2). The MISC_A tumor mean coverage is 60.5×, but MISC_A tumor–based HLA typing results are consistent with normal sample (254× mean coverage)–based HLA typing results obtained using ATHLATES and Polysolver (Fig 2).

Mutations in the HLA gene regions have been previously described by Shukla et al^26^ with a suggestion that somatic mutations in HLA class I genes are likely positively selected during cancer evolution and contribute to immune evasion by cancer cells. The study by Shukla et al^26^ analyzed 7,930 tumor-normal pairs and found somatic nonsilent mutations including loss-of-function mutations in 3.35% of the tumor samples studied. For personalized neoantigen peptide vaccine discovery purposes, it is suggested to perform HLA typing using tumor tissue as somatic mutations may be present in HLA genes in tumor cells,^54^ which may affect the HLA type deduced. Somatic mutations in the HLA genes in the tumor may also be the source of variation in HLA typing calls from tumor and normal tissues.^26,54^ We did not find such an effect of somatic mutation causing variation in HLA typing call in our analysis cohort.

In a lung cancer study, loss of heterozygosity of HLA alleles was shown to be contributory to immune escape and cancer tissue evolution.^27^ Complete or partial deletion of one copy of chromosome 6 is found to be present in many cancer types^55^ including GBM and ESCA. Entire chromosome 6 copy deletion should indicate complete loss of heterozygosity of HLA class I and HLA class II genes located on the lost chromosome 6 copy. Along with accurate HLA allele type detection, the HLA typing software tool should be capable of detecting loss of heterozygosity when cancer tissue sequencing data are used. None of the four HLA typing software tools used in the present study were designed to detect HLA gene loss of heterozygosity; still, Polysolver was the only software package which could consistently detect the loss of heterozygosity in HLA calls from tumor samples especially those with high tumor purity (Figs 2 and 3). The other three software tools could not consistently predict the homozygous status of HLA calls which were the outcome of HLA loss of heterozygosity in the tumor samples. The effect of tumor purity on the detection of the loss of heterozygosity by the HLA typing software tool was evident in the HLA typing analysis. In tumors with low tumor purity, heterozygous HLA allele genotypes were predicted even in the presence of HLA allele loss of heterozygosity or loss of one copy of chromosome 6 (Figs 2 and 3).

The results from the present study indicate the significance of integrative genomic analysis of the status of HLA alleles in tumor tissues and also highlight the need for specialized software tools which can perform such analysis along with HLA genotyping. Somatic missense or loss-of-function mutations, microdeletions within the HLA region, or the loss of one copy of chromosome 6 could potentially act as immune escape mechanisms, thereby promoting cancer growth and resistance to immunotherapy.^56^ The mutant or lost alleles may not be available inside the cancer cell to functionally present the neoantigen peptide fragment to CD8-positive T cells. The results suggest that the HLA alleles found to be mutant or lost should be excluded from the patient HLA genotype being used in the personalized neoantigen peptide prediction pipeline. The inclusion of only intact HLA may potentially increase the efficacy of PNPVT, but this needs to be proven experimentally. This will not only prevent additional expenses associated with those peptides predicted to bind to lost HLA alleles but also enable a more targeted immunotherapeutic approach, concentrating on intact HLA alleles. The use of a subset of functional HLA genotype, in turn, will be helpful to enhance the precision of PNPVT. Integrative genomic analysis of HLA genes will be useful to identify a subset of high-confidence HLA genotype. The neoantigen peptides can be predicted against only the high-confidence subset of HLA alleles which are found to be intact in the cancer cells.

In the current clinical practice, the normal tissue–derived HLA genotype is being used for the prediction of personalized neoantigen peptides to be used as vaccines. The results in the present study point out that this clinical practice should be modified to include the analysis of the status of HLA genes in the cancer tissues in the clinical workflow. The HLA genes affected by somatic mutation, which potentially affect the HLA structure and function, should be excluded. The lost HLA genes and HLA genes located on lost copy of chromosome 6 should also be excluded from the neoantigen peptide discovery pipeline. Though with caution, these workflow changes should be incorporated only after assessment of the impact of somatic HLA gene changes on PNPVT outcomes in a larger cohort for the concerned cancer type. In addition, tumor tissues with high tumor content provided better results for mutation, HLA LOH, and chromosome 6 copy loss analysis, so cancer tissues with high-tumor purity samples should be used for exome sequencing and RNAseq.

## Data Availability

A data sharing statement provided by the authors is available with this article at DOI https://doi.org/10.1200/CCI-24-00174.
